# The Nerves

**Published:** 1834-07-01

**Authors:** 


1. A Treatise on Diseases and Injuries of the Nerves. By *Joseph Swan*. A new Edition, very considerably enlarged. Octavo, pp. 356—10 Plates. London, 1834.
2. On the Reflex Function of the Medulla Oblongata and Medulla Spinalis. By *Marshall Hall*, M.D. F.R.S. &c. &c. From the Philosophical Transactions. London, 1833.
3. A New Exposition of the Functions of the Nerves. By *James William Earle*. Part I. pp. 195. London, 1833.

The three works, or portions of works, before us, contain very nearly six hundred pages. They form but a tithe of what has been written and said, of late years, upon the nervous system. The discoveries of Bell have brought into the field a host of inquirers, each of whom has his theory or his facts with which to settle some disputed point, or illuminate some mystic doctrine. Many, like Majendie and Flourens, endeavoured to dazzle and to captivate by their sleight-of-hand experiments and outré discoveries. They have boxed the compass in the cerebrum, and no one knows how the needle points. Others have laboriously contrived to throw confusion upon doctrine and on fact, and endeavoured to extinguish the lights we possess, without substituting even a glimmer in their place. A few, and but a few, have marched slowly on in the path of induction and of caution, and have offered steady and consistent, if not brilliant information.

The first work on the list is of the latter nature. We commenced a notice of it in our last, and we promised to complete that notice in the present Number. We cordially redeem our pledge. Although it embraces physiological inquiries, its general character is pathological. Mr. Swan presents us with what few authors have attempted—a connected history of the morbid changes that occur in the organic tissues of the nerve, their symptoms, and their treatment. He does not pretend that the Treatise is free from deficiencies and defects, but he trusts that his attempt will be attended with the beneficial consequence of stimulating investigation, and affording an impulse to the observation and diffusion of facts.

The work of Mr. Earle, a relation of the gentleman who holds the office of surgeon to Bartholomew's Hospital, is more of a speculative turn than the preceding. The great aim of the author would seem to be, to run-a-muck at the doctrines of Dr. Wilson Philip.

The paper of Dr. Hall, like others from the same writer, is ingenious, and will occupy our attention. It displays a physiological aspect.

It would be tedious and unprofitable, if not impracticable, to endeavour to amalgamate, in one connected article, works of so various and so opposite a character. We shall, therefore, present such a notice of each in succession, as our limits and their merits will justify or permit.

Of sixteen chapters, composing the work, of Mr. Swan, two were noticed in the last Number of this Journal. The remainder are devoted to the following subjects :—Painful affections of the nerves in general—painful affections in particular nerves—inflammation of nerves—ulceration of nerves—tumors in nerves—divided nerves—partially-divided nerves—effects of ligatures on nerves—compression of nerves—diseased appearances of the spinal canal—injuries of the spinal chord—diseases of the nerves of the senses—diseases of the sympathetic nerve—and tetanus.

### I. Painful Affections of Nerves in General.

We cannot refrain from extracting a remark of Mr. Swan's, in reference to the conclusions derivable from experiments. Unless the experimenter be possessed of what he frequently is totally devoid of—cautious scepticism and sober judgment, he will often draw the most erroneous and absurd deductions from what appears to be the most exact and most demonstrative method of investigation. The records of modern physiology afford too maay specimens of this description.

" Experiments (says Mr. Swan) for determining the sentient qualities of nerves have not been so satisfactory as to have carried conviction to the minds of unbiassed persons, for when animals of the same species have been subjected to the same treatment, one has expressed the keenest suffering, while another has been altogether unmoved. But observe what takes place on the performance of an experiment. Incisions are made through sensitive parts ; these are forcibly retracted for allowing the separation of the specific nerve from its surrounding connexions, and the state of the wound is momentarily varying, so that when the nerve itself is irritated, it is impossible in many instances for the most candid observers to form a correct judgment." 3.

Mr. Swan fills several pages with arguments, to shew that the state of the sanguineous system in general, and that portion of it appropriated to the nerve, must exercise an important influence upon the latter. Perhaps the nature of the subject may offer a satisfactory excuse for the vagueness of the reasoning. At all events we must pass it by, in order to attend to more obvious facts. It would appear that pain, affecting the nerves, frequently occurs in irregular paroxysms, or even at regular periods. In a case of popliteal aneurism, for which the femoral artery was tied by Mr. Swan, the latter phenomenon was noticed. For five weeks previous to the operation, violent pain came on in the leg about half past ten o'clock every night, and went off at two in the following morning. It appeared to be seated in the sciatic nerve, and was never felt after the operation. In a patient who died of apoplexy, an ulcer placed over cutaneous nerves, near the ankle, was affected with the most excruciating pain at the same hour every morning.

Mr. Swan approaches, with less timidity than moderate caution might occasion, two subjects of no inconsiderable difficulty—the cause of periodical changes in the functions in health and in disease—and, the primary condition of the nervous system which gives rise to fever. In the meshes of these questions we leave Mr. Swan, and again proceed to obvious fact—the influence of the digestive organs in the production of painful sensations in various portions of the body. There are few physicians or surgeons in good practice, who have not frequently observed the occurrence in question. Instances have been related in which pain was thus occasioned in the cutaneous branches of the fifth—in the nerves of the hand and of the foot—in the shoulder—in the perinæum, and in other parts. On the other hand it is well known that local irritation of a nerve will induce disorder of the digestive organs. Dentition affords many instances of this description. Mr. Swan relates some facts which are worthy of attention. Mr. Pearson, for example, has published the case of a gentleman who had an issue in the thigh which produced both vomiting and deafness, and he has also stated that both these symptoms were entirely removed by healing the ulcer. Bichat* says in neuralgic paroxysms there are often spasmodic vomitings, increased actions of the heart, &c. The same symptoms may be produced in making experiments. Thus, in acting on the nerves of the inferior or superior extremities, by irritating them in any manner after they have been exposed, Mr. Swan has often produced vomitings, or convulsions in muscles not supplied by the nerves he irritated. Mr. Wardrop has related a very interesting case. A woman pricked the fore finger of her right hand with a gooseberry thorn, the two first phalanges were very painful, and so acutely sensible as not to endure being touched. After having suffered very much for twelve months, the finger was amputated, and she got completely well.

"The nervous paroxysms," says Mr. Wardrop, "usually attacked her two or three times a day, and one of them always came on at the time of her rising out of bed. During these attacks the pain extended along the finger to the back of the hand, and between the two bones of the fore-arm, darted through the elbow joint, stretched up the back of the arm to the neck and head, producing a sensation at the roots of the hairs as if they had become erect. To these feelings succeeded a dimness of sight, and the pain afterwards went suddenly into the stomach, followed by sickness and vomiting. She had constantly the feeling of a lump at her stomach, and always vomited after taking food or drink. Her flesh too was much wasted, and she had become extremely pale." 19.

In half an hour after the operation the patient felt as well, with the exception of a slight pain in the stump, as she had done previously to meeting with the accident.

Mr. Swan glances timidly at a curious question—how pain is conveyed from one part to another. The enumeration of a few particulars will perhaps serve rather to expose our comparative ignorance, than to shew the extent of our information on this subject. When pain is conveyed from the viscera to the different parts of the body, or from these to the viscera, Mr. Swan observes that the sympathetic nerve and the par vagum form the medium of communication. But we cannot be assured that the spinal nerves and the medulla take no share in the office, nor can we determine what part is enacted by any. The bladder, uterus, and rectum are supplied by plex-

* Anatomie Generale, tome i, page 186.

uses formed of the combination of spinal nerves with branches of the sympathetic. Now, affections of the bladder cause pain at the end of the penis, or even in the sole of the foot—and pains in different parts of the extremities are experienced in diseases of the uterus and rectum.

In some cases pain has been exclusively experienced on the *side* of the body on which the injury was inflicted. Mr. Swan alludes to the instance of a lady in which this occurred. But females are so frequently affected with anomalous pains of all descriptions, that we fear it would be dangerous to generalize on their peculiarities.

Sometimes the pain does not reach far beyond the affected part, but produces a great weakness of all the muscles of the limb, with wasting, and an imperfect generation of heat. In some cases of tumor in the nerves, the brain has been affected as in epilepsy; and from a violent cause the irritation of a nerve may be extended to the brain, as in the case of a general officer, related by Dr. Hennen,* in which some of the axillary nerves had been included in ligatures after amputation of the shoulder-joint, and pain was felt in the course of the brachial nerves, just as if these had existed, whenever the ligatures were gently pulled; but on one occasion only, when these were forcibly extended, his head became suddenly affected. Diseases of the brain will produce pain in particular nerves; and, on the other hand, violent and long-continued pain in the nerves may produce disorder in the brain.

Mr. Swan observes that we must not lean too much to the idea that nervous affections imply some peculiar constitutional defect. We must always endeavour to discover the cause of the affection, and, having discovered it, we may possibly remove it. The connexion of morbid excitement of the nervous system with debility is one which should ever be retained in view.

Mr. Swan offers some judicious reflections on the treatment of neuralgic maladies. It is not surprising that his observations present little novelty, and throw no light on the management of an intractable class of disorders. Our knowledge of his practical abilities would have led us to anticipate, what indeed is actually the case, that Mr. Swan would discountenance that empirical employment of drug after drug, which is far too prevalent, and that he would forcibly impress upon his readers the necessity of endeavouring to discover the general or the local cause of the complaint in each particular instance. It is chiefly in this power of analysis and discrimination that the able practitioner is distinguished from the dull and plodding routinist.

Mr. Swan admits the fallaciousness of those hopes which were once entertained respecting the division of the affected nerve. He appears to prefer the excision of a portion, as being more effectual. Of course when the cause of the neuralgia is deep-seated, neither this nor division can possibly succeed. But in some few instances, and probably they *are* few, cutting off the communication between the part of the nerve which appears to be the seat of pain, and the cerebrum, may be useful—and, in such, we need not say, that removal of a portion of the nerve must be more efficient than a mere division, which permits of a subsequent reunion.

---

* Military Surgery, p. 191.

### II. Painful Affections in Particular Nerves.

In our notice of this volume we do not pretend to present a strict analysis, for there is necessarily much that is familiar to the well-informed part of the profession. But there are many facts and observations brought forward by the author, which are either new, or arranged in such a manner as to merit our attention. It is our object to select such matter as may seem valuable, and transfer it to our pages in as close and compact a form as its nature will permit.

Passing over some general remarks on intermittent pains, we may extract the following brief enumeration of the causes of tic douloureux. They are, of course, only the condensed expression of many individual facts.

"Tic douloureux in the nerves of the head and face may arise from a spasm of the nerve through sympathy with a distant part; from inflammation of the periosteum and enlargement of the bone contracting the size of the foramina through which the nerves pass, and producing compression or distension; from exfoliating portions of bone exciting irritation and sometimes producing ulceration; from decayed teeth; from spiculæ of bone irritating the brain; from too much vascular action in the brain; from tumours pressing on the brain and the origins of the nerves; from too great vascular action in the painful part; or from connection with an inflamed part as in iritis." 40.

From this enumeration, and probably it is incomplete, the practitioner may infer how indispensable it is to investigate each particular case—to discover what is wrong—and to set that right. But with many this very caution becomes an apology for routine, and an excuse for ignorance. If, say they, the great art is to treat symptoms, why plague ourselves with reading, why lay upon our backs a load of learned and of useless lumber? Such observations we have often heard from the mouths of those half-witted individuals, who affect to despise the accomplishments of learning in their more enthusiastic and more scientific brethren. But these miserable hacks, these petty dealers in purging and in sweating overlook one very important circumstance. They forget that learning, rightly understood, consists in the addition of the experience of others to our own. It is not the wide acquaintance with theories—it is not the ability to cite author after author, name after name, to support some good, or to bolster up some stupid notion—it is not the knowledge of what every one has said or done on every subject since the æra of Hippocrates—all this may be the learning of the gownsman or the pedant, but practical medical learning it is not—genuine philosophical knowledge it cannot be. No, learning in its true sense consists in the accumulation of facts—its right application in the proper use of them. The experience of one man is totally inadequate to store his mind with sufficient facts, to enable him to take a comprehensive view of any subject that presents itself. But, if gifted with the power of distinguishing true learning, and applying it, he renders the observations of others subservient to his own, and extracts the good ore from the earth and the rubbish that inclose it.

The disease before us, tic douloureux, may form an illustration of these remarks. The routinist would either treat symptoms exclusively, or blindly resort to empirical remedies. If his instinct prompted to the former, it would be utterly impossible for him to be aware that, in some cases, tumors on the brain had given rise to those symptoms, whilst, in others, a carious tooth, and, in others, an exfoliation of the alveolar process of the maxillary bone had been proved, by observation, to occasion them. He might, therefore, prescribe his purgatives or his narcotics, according as the bowels seemed deranged or the nervous system was excited, while the cause of all disturbance remained untouched. Or, if he adopted the empiric method, he might order arsenic or iron, in cases in which there was vascular action in the brain, or in which some source of irritation existed, that tonics or stimulants would render more injurious. But the man possessed of true practical learning would attend to symptoms, and would be ready to apply what we may denominate empirical remedies, at the same time that his observation of the former was directed, and his administration of the latter modified, by his accurate acquaintance with recorded facts, that is, with the circumstances which dissection and observation had shewn in other cases, besides the few he had personally witnessed, to cause or to accompany the disease before him.

We must not lose sight of Mr. Swan. He relates some cases which are worth remembering. In one, a young gentleman had excruciating pain in the face, which had existed for two months, resisted many remedies, and followed a blow, which broke one of the middle incisive teeth of the upper jaw. There did not seem to be any obvious cause for the affection. Mr. Swan cured him by wine and bark. In another case, a gentleman suffered for months from excruciating pain at the back of the head, which succeeded a meal on hashed hare, which had stood in a brass pan covered with verdigris. He was much emaciated. He had employed in vain a variety of remedies. Mr. Swan prescribed a blister at the back of the neck—thirty drops of laudanum to be taken in the morning, when the pain was usually most severe—wine and malt liquor. This plan effected a cure.

"I have seen several severe cases of this complaint, which no means seemed to lessen; and it was only when the teeth on which the disease depended had been extracted that the symptoms yielded. Many people are unfortunate in being obliged to lose several teeth, before the right one is discovered; but the diseased one may generally be known by striking the teeth with a piece of iron, or by feeling between them with one of the bent instruments, used for filling them when decayed." 42.

In one case, where the patient suffered the most excruciating pain in the tongue and throat, and could only swallow with the greatest difficulty, a small portion of the alveolar process irritated the tongue. Mr. S. removed this with his finger. The pain immediately ceased, and did not return.

Mr. Swan observes that it is not unusual, in diseases of the lungs, for the functions of the brain to become disordered, and to be accompanied by severe pains in the head and face, resembling tic douloureux. If the perspiration has been checked by acids, &c. says Mr. S. it ought to be restored, as the best way of relieving the brain and the affected nerves. He relates an interesting case, communicated to him by Sir A. Carlisle, in which bleeding removed the complaint. A lady, aged eighty years, had tic douloureux in the suborbital branch of the fifth pair of nerves. The pulse was very full, therefore sixteen ounces of blood were taken from the arm. Three days after she thought herself relieved, but as the pulse continued still very full, twelve ounces more of blood were taken away. Three days after the last bleeding, she was bled again to sixteen ounces. She was now entirely free from pain, and never had any return of it, and died two years after this period of apoplexy.

Practitioners should remember that, in cases of this disease in advanced life, great caution should be used in the exhibition of stimulants and tonics, the brain being frequently affected, and apoplexy liable to supervene.

Mr. Swan gives a case, in which a gentleman suffered from pains in various parts of the body, but most frequently in the limbs, which resembled tic douloureux in violence. They were sometimes confined to a small spot in one leg, sometimes in the other, or in the arm, or shoulder. When situated in the latter part, the patient was usually very sallow. The complaint arose from a disordered state of the digestive organs. By the use of blue-pill, and aperient and tonic medicine, he was always very much relieved, and for some time remained quite well. When the pain was in the lower extremities, the cause was generally a collection of fæces in the larger intestines, and he was relieved by purging.

Mr. Swan observes, and the caution should be treasured in the minds of practitioners, that when painful affections of the nerves are complained of, especially in the extremities, the condition of the intestines and of the genital and urinary organs should be carefully investigated. Diseases of the alimentary canal will produce partial affections of the nerves of the limb, and sometimes of a single finger. Pain does not always affect the nerves immediately connected with those of a diseased organ, but sometimes such as are at a distance. One gentleman had generally pains in the fingers whenever he had a motion; another, at the backs of the fingers in evacuating the bladder, when much distended. In the case of a lady, who suffered from disturbance of the digestive organs, with palpitations of the heart, pain in the course of the left ulnar nerve ceased after a severe affection of the uterus. Our author states that, after accidents which have affected the muscles or the nerves, pain and weakness will continue for a long time, and the general health become impaired. In several of these cases, the subcarbonate of iron, taken in large doses, has strengthened the system, and allowed the local morbid irritation to subside.

Mr. Swan concludes the chapter by some observations on those painful affections of the back, unconnected with actual spinal disease. We shall have an opportunity of directing attention to this subject in our next Number, and we, therefore, proceed to the third chapter of the work before us.

### III. Inflammation of Nerves.

This section is brief, and its contents are proportioned to its brevity. They may be simply stated to amount to little more than this:—That acute or chronic inflammation, in parts surrounding nerves, may implicate the latter —that the nerves of stumps not unfrequently become affected with chronic inflammation, occasioning enlargement of their ends—that the nerves in the neighbourhood of diseased joints are also apt to become enlarged—and that sciatica appears to be a genuine idiopathic inflammation of the great sciatic nerve.

But there are some particular circumstances or facts, at which a less hurried glance is necessary.

Thus, Mr. Swan relates a case in which, after suppuration beneath the palmar fascia, great heat and a pricking pain, with diminution of sensation, remained for some time in the three large fingers, which were bent down to the palm. The patient gradually recovered. He alludes to Dr. Hennen's statement, that, after gunshot injury in the vicinity of a nerve, secondary paralysis frequently takes place, in consequence of inflammatory action extending to it, or tumefaction pressing on it. Œdema of the extremities is another consequence of a similar condition. Mr. Swan himself details an interesting case of enlargement of the median nerve, from an injury to the wrist. We must extract the case.

*Case.* " David Franklin, aged twenty-two years, was admitted into the county hospital about the middle of October, 1820. He said that, seven years before, he was holding a horse, with the halter tied round his hand, when, the horse running back, the wrist was injured, and became immediately bent. The part was violently pulled after the accident, and thereby further injured. He had great pain in the wrist and palm of the hand ever after, and a slight pain at the back of the hand. The skin at the back of the hand was injured, and was continually ulcerating. The thumb and three fingers were always bent towards the wrist, and could be extended only in a very small degree, and the sense of touch was lost. As the hand was quite useless, and the source of much inconvenience, I amputated it, and the part soon healed.

On examining the hand, I could not perceive any other alteration in the carpal joints, except what would arise from their being kept constantly bent, and it did not appear that any serious injury had been inflicted on them. The muscles in the anterior part of the forearm had contracted a permanent shortening. The median nerve, where it passes under the annular ligament, was much enlarged, and its natural connexion with the sheath of the tendons of the flexor muscles of the fingers thickened. Several of the digital nerves, towards their termination, had a gangliform enlargement." 61.

Mr. Swan next adverts to Mr. Langstaff's paper on the healthy and morbid conditions of stumps, and to the well-known case which he relates, of a female who twice underwent amputation, for excruciating pain dependent on a morbid condition of the nerves. On examining, after the second amputation, the stump which had resulted from the first, the median, radial, and ulnar nerves were found remarkably large, the extremities of the two latter being greatly increased by deposition of organized lymph. The second amputation was successful, and we may venture to remind our readers, that this success was probably due, in a very great degree, to the precaution adopted by Mr. Langstaff, viz. drawing out each nerve to the extent of half an inch from the surface of the stump, and then cutting it through.

On the treatment of inflamed nerve nothing need be said. The principles which must direct it are obvious enough.

### IV. Ulceration of Nerves.

This chapter is more ingenious than satisfactory, and perhaps the amount of really useful information contained in it is small. Our author appears unable to offer much that is certain with respect to the ulceration of nerves. He relates no case in which this can be looked on as an idiopathic or peculiar lesion. An instance quoted from Morgagni is the most direct and most to the point of any. The case in question was one of aneurism in the right groin, which extended backwards so much as to produce an ulceration of the sciatic nerve.

"In the last month the pains became most severe, not only in the tumour, but sometimes beneath the internal malleolus, in which place only, violent as the pains were, the foot was sensible, being in every other part deprived of feeling and motion. In all this month, he neither had an interval of ease, nor any sleep until his strength failed; then for some days he lay half asleep, and so died.

On examining the limb, the nerve was so much eroded, that a few fibres hardly remained, by which the superior and inferior parts were joined together." 84.

The chief aim of Mr. Swan, in this division of his work, appears to be, to account for the sufferings which some ulcers cause, and to suggest the propriety of removing a portion of the nerve or nerves that are implicated in such ulcers. He details, at some length, the particulars of three cases of this description, in one of which the operation was performed, though with only a partial degree of success. In the first case, a fungous ulcer was connected with a carious hollow in the tibia. The sciatic nerve and many of its branches were enlarged. The branch of the anterior crural, accompanying the saphena vein, was at one part nearly surrounded by the ulcer, and in two others firmly united to the adjacent parts. The peroneal nerve and its branches were enlarged. The dorsal branch was at one part surrounded by the ulcer, to which an inch and a half was firmly united; an inch of this appeared rather smaller, and in a state of ulceration, and in one place nearly divided. The anterior tibial nerve was also united to the surrounding parts, and in one place so compressed by the enlarged tibia, as only just to admit of being recognized.

In another case, a fungous ulcer existed on the outside of the left leg. The pain was extremely severe. Branches of the peroneal nerve were found to be involved in the base of the fungus.

In the the third case, excision of a portion of the affected nerve was performed. The ulcer was situated on the left leg, beginning about its middle and reaching nearly to the instep; it extended across, from beyond the inner edge of the tibia to the fibula. The pain was violent, and extended up the outside of the leg to the ham, and from thence to the back. Mr. Swan excised about an inch of the peroneal nerve, at the ham. The patient was greatly relieved after the operation, but he still suffered occasionally from the connexion of the saphena nerve with the ulcer. The latter of course continued —the patient wasted, and amputation was performed. The tibia was found affected with caries and necrosis. The saphenus nerve was much enlarged, and was connected with the ulcer. The sciatic and the peroneal nerves were enlarged also. At the place where the latter was divided, one new branch, if not more, went from the upper portion to the anterior tibial nerve.

It is probable, from a candid review of these cases, that the operation of removing a portion of a nerve will be applicable to few instances of painful ulcers. For, in an early stage, the ulcer may admit of cure, and the pain be in consequence removed, without the necessity for an operation; and in the latter stages, when an operation is more indicated, the disease has involved so many tissues, that amputation is required to preserve the limb or life. At the same time, it would be proper to keep Mr. Swan's suggestion in view, as a case might occur adapted for a trial of it; and the cases he relates are valuable, as shewing the exact condition of the nerves in many very painful ulcerations.

"In people advanced in life, the teeth of one jaw are sometimes entirely lost, so that those of the other are apt to press very much on the soft parts of the mouth, and produce slight ulcerations, which sometimes cause very violent pain, exactly resembling the tic douloureux; therefore in all cases which will not yield to the usual remedies, the mouth ought to be carefully examined, as the symptoms depending upon an ulcer produced in this manner can only be cured by extracting the tooth.

Sometimes small portions of the alveolar processes will exfoliate and remain attached to the gum, producing violent pain in the tongue, and rendering the act of deglutition very painful; therefore, when the nerves of the tongue are affected, it is right to feel along the inside of the gums with a finger, that any rough substance may be removed. In all other painful affections of the face, similar examinations should be made, as incrustations of tartar may, by pressure, produce ulcerations, and be the cause of unyielding symptoms." 85.

The fifth chapter is devoted to the consideration of tumors in nerves. This was noticed in our last Number, and we, therefore, pass on to the subject of the sixth chapter.

### VI. Of Divided Nerves.

The symptoms occasioned by injuries of the nerves, though occasionally very violent, are too capricious to allow of any opinion being formed, à priori, respecting their severity. When a nerve has been divided, and the external wound has healed by the first intention, very little pain is usually experienced. But an open ulcer, connected with a wounded nerve, is generally very painful, and sometimes produces very violent symptoms; so that when a nerve has been divided, it is a matter of the greatest importance to produce union of the parts about it by the first intention, because then the divided extremities of the nerve escape many sources of irritation. It is necessary to remove as much as possible of the coagulated blood, and every thing that can act as an extraneous body. If the external wound has healed, and there are marks of inflammation about the cicatrix, the nerve is frequently affected with severe pain, which is generally aggravated by any motion of the part. Rest, leeches, and evaporating lotions are the best means to be employed.

*Case.* A man had a fungous tumor on the lower lip, in which he suffered excruciating pain, resembling tic douloureux. Mr. Swan cut out the diseased part, and the wound healed well. In about a week the patient returned. The upper part of the cicatrix was rather redder and more swollen than it should be, and was still the seat of excruciating pain. Mr. S. ordered two or three leeches to be applied to the lip, and prescribed appropriate remedies for a furred tongue and disordered stomach, under which the patient laboured. These remedies removed the complaint, and several years afterwards the patient remained quite well.

Mr. Swan relates a case, displaying the advantage and necessity of attention to the state of the digestive organs, in all diseases and injuries of the nerves.

*Case.* "Miss Carr, about thirty years of age, in cutting some bread, wounded the skin on the inner side of the thumb, as near as possible to its extremity.

She said there was very little bleeding at the time. When I first saw her, the wound was quite healed. She could not put her hand behind her. She had numbness in the thumb, and occasionally pain in the outer side of the forefinger, the pain extended as high as the elbow. She had pain in the head before the accident, and now her digestive organs are very much disordered. She had fomentations of poppies applied to the hand, and a poultice made of the same to the thumb. She took five grains of blue-pill every other night, and an aperient mixture with magnesia. The pain began to abate in a few days, and gradually got less, until it entirely left her." 103.

When a nerve has been divided, and the wound does not heal by the first intention, but inflames, violent pain, and frequently spasms are produced, from the communication of the inflammation to the nerve. Mr. Hunter has shewn that a wound of any part, made internally, may heal by granulation, independent of any secretion of pus. Mr. Swan has seen this process, in the healing of a divided nerve, in a dog, who did not appear to suffer much pain. Neither is much pain experienced when divided nerves heal by the effusion of coagulable lymph.

"Nerves connected with external wounds granulate like other parts, as I once had the opportunity of observing in the leg of a boy, that was amputated some time after a compound fracture, when the soft parts were in a state of ulceration. Although the result has been nearly the same in all the experiments I have made, yet, in some few, Nature has been backward in perfecting the restorative process. And even when experiments have been made on both the posterior extremities of the same rabbit, the divided nerves in the two limbs have now and then exhibited very different appearances; in one, the healing process has been going on properly; in the other, hardly any attempts have been made to begin it; the usual increased vascularity has been wanting, and consequently the deposit of coagulable lymph; and thus I conceive it may be in the human subject, for, in some instances, the union never properly takes place, as the patients are ever after either deprived of sensation, &c. or exposed to unpleasant effects from cold, &c. in the parts beyond the divided nerve." 105.

In a case of fracture of the thigh-bone immediately below the trochanter, in which the patient suffered more from pain than is usual in such injuries, the sciatic nerve was found to be partially lacerated. Mr. Swan is of opinion that such accidents are more frequent than is commonly supposed.

### VII. OF PARTIALLY-DIVIDED NERVES.

Mr. Swan observes that, when a nerve has been wholly divided, each portion immediately retracts, so as to leave a considerable space between them. But when only a partial division has taken place, the divided portions retract in the same manner, but not in so great a degree, and leave a space, whilst the undivided portion remains of the same length as before the division. Under these circumstances, the undivided fibril or fibrils must be stretched, and this stretching *should* prove a source of pain and irritation. Yet facts would rather seem to shew that it does not; for, from the experiments which Mr. Swan has made in partially dividing the nerves of animals, it does not appear to him that they suffer more than when a nerve has been wholly divided.

Mr. Swan devotes some pages to the consideration of wounds of nerves in the operation of venesection. That this must occasionally happen, no one acquainted with anatomy can deny, but that the bad effects have been greatly exaggerated we cannot for an instant doubt. Many of the cases related as examples of this accident appear to be only instances of those hysterical pains to which young women are prone, which frequently follow trifling operations, and as frequently occur independently of any. However, Mr. Swan rather leans to the opinion that the accident is frequent, and appears to have met with many examples of it. We cannot say that we ever witnessed one satisfactory instance, although it may be readily believed that we have seen a fair number of persons bled. Mr. Swan attributes a large share of the mischief to inflammatory action in the wound.

"I have very little doubt but by far the greatest number of injured nerves in venesection is made troublesome by using the arm too soon, and bringing on inflammation; for I have never seen any bad consequences in those patients who have been so ill as to be unable to do any thing.

Whenever a person has complained of very acute pain on the opening of a vein, great care ought to be taken to close the wound well with a linen compress and bandage, and to keep these continually on the orifice, and at the same time to confine the arm in a sling until the wound is perfectly healed." 115.

He relates a case to prove, that if the external wound heal by the first intention, the injury of the nerve may be of little consequence. He alludes to the supposition that the injury of a nerve in venæsection is always a partial division, and that a complete division is necessary to effect a cure. This notion is obviously so vague, so unsupported, and so contradicted by facts that it does not require particular refutation.

Mr. Swan details some cases to shew the serious symptoms that may supervene after such injuries. Omitting one from Bonetus, we may introduce one communicated to our author by Dr. Wilson, of Grantham, because it displays the general characters of cases of this description.

*Case.* "I was desired," says Dr. Wilson, "to visit Mr. B.'s housekeeper, at ——, I found my patient in strong convulsions, and held upon the bed by several assistants; her hands were strongly clenched, and she was struggling greatly: she soon after became comatose. I was informed that she had been let blood two days before by a gardener; that she complained very much of the arm where she was bled, and of a pain shooting from thence to the shoulder. I examined the orifice of bleeding, which was in the median vein; it had not healed, was somewhat inflamed, and a thin liquor oozed from the lips of the wound. While I was making this examination she became again strongly convulsed, as I supposed, from the irritation I had caused. With a view to interrupt the communication from the diseased point to the sensorium, I applied a tourniquet above the part: a remission of the spasms soon followed, and I administered an anodyne; but the convulsions, after a short interval of ease recurred as before, and the application of the tourniquet was again made without any good effect. As I had no doubt that the cause of the disorder was an injury of a cutaneous nerve in the operation of venesection, I determined to endeavour, by a transverse incision, to divide the nerve above the injured part, and to destroy its connexion with the sensorium; I therefore made an incision while the convulsions were most violent, of about an inch in length and small depth just above the orifice: no mitigation of symptoms was perceived; but on making another incision above the former one, somewhat deeper and longer, she cried out immediately, to the astonishment of the attendants, 'I am well, I am quite well, I can move my arm;' which she began to move, and continued to do so with great delight for some time in various ways. She had no return of the spasms, and very soon got well." 119.

It is worthy of remark, that the subjects of the majority of these cases are females. We cannot but suspect, which we do however with humility, that the lady in the last instance gave way to that peculiar habit of her sex—excitability of the nervous system on comparatively slight occasions. It is curious, at all events, that the relief from division should be so instantaneous. An uncharitable person would hint that the knife might possibly have proved disagreeable.

Mr. George Bell has related a nearly similar case, in which he adopted a practice which will find few imitators or admirers. He applied two ligatures upon the vein, and removed the portion contained between them. We need not point out the obvious dangers attendant on such an operation.

Mr. Swan supposes that in the wounds made in venæsection, it is frequently a very small filament that is disturbed, which in some instances may be connected with the edge of the wound; and he imagines that the unfavourable symptoms may be removed by nipping up the portion of skin forming the lips of the wound, and removing a very little with the blade of the knife held horizontally. He mentions the particulars of a case illustrative of these suppositions, but it does not appear by any means satisfactory. Another case is quoted from Sabatier's Médecine Opératoire, in which uncontrollable trembling of the leg and thigh succeeded a stab at the inferior and inner part of the left thigh, in the course of the saphena vein and nerve. The patient ultimately recovered without any operation. Mr. Swan relates a striking instance of the melancholy severity and pertinacity which these affections occasionally manifest.

*Case.* Mrs. E. æt. 40, received a cut on the inside of the first phalanx of the left thumb. Immediately after the accident she felt a numbness in the arm, and a sense of fulness, as if the skin would burst; these sensations continued for a fortnight, and the wound healed very well. At the end of this time, violent pain came on, when a tremulous motion could be seen in the part which it occupied. The pain was termed startings, or spasms, by the patient, and was felt in different ways, but the muscles were not affected. These spasms were felt all over the body, though they were by far the most frequent in the upper half of it. She frequently felt a great heat in the chest and abdomen, but most particularly in the latter, and the same startings as in other parts of the body. The sensations were sometimes as if the flesh was pinched with hot irons; sometimes a great heat, as if hot water was poured down her back; sometimes she had frequent shakings of the whole body, which were unattended by pain, and were most relieved by drinking hot water. The spasms were not confined to the left arm, but she had them at the same time in the right, and frequently in the right when she had none in the left. The fore-finger was as painful as the thumb, and if any thing touched either of them the spasms were produced, which continued many days. She had a good appetite, her bowels were confined, and her tongue furred but she had no thirst.

Sedatives and antispasmodics seemed to do her most good, though nothing was productive of much benefit. At the end of six months the spasms were less frequent, but were reproduced if the thumb or forefinger was moved or touched. Succeeding years brought each some mitigation of her sufferings, but even after seven had elapsed, extreme susceptibility of any impression still remained in the thumb, and the forefinger had not recovered its natural condition. Lifting a weight, or using the right arm much, always produced sensations as if needles were running into it, and attempting to use the fingers of the left hand, as in knitting, produced giddiness. She continued to be affected with the spasms in every part of her body till her death, which happened nine years and six months after the occurrence of the accident, when she seemed to die worn out.

Mr. George Bell has published a case in which violent nervous symptoms followed a wound on the radial side of the thumb, half-way between the first and second joints. After a variety of means were employed without avail, amputation proved successful.

Mr. Wardrop has related a case in some respects similar to the preceding one, and in which he relieved the patient by an incision made beyond the wound. There was more febrile excitement in this case than is usually observed, and it is most probable that it was very much occasioned by intemperate living, which also produced inflammation of the injured nerve, and the other symptoms.

Passing over a very long case detailed by Mr. Swan, we shall merely pause to extract his conclusions respecting the treatment of these affections.

" From experiments on animals it appears, that in whatever way wounds of the nerves are made, they are repaired by nature alone. It becomes then necessary to inquire under what circumstances the interference of a surgeon is requisite, when the nerves have been injured in the human species.

In bleeding, it is undoubtedly proved that the nerves sometimes suffer from a wound by the lancet; in many instances there have been violent symptoms, but in course of time these have subsided: it is therefore right, when this accident has happened, to wait for a while, and try by palliatives to assuage the violence of the pain and other symptoms that may occur. But if the irritation be so great that convulsions have come on, as in a preceding case, and threaten the patient's life, a similar operation should be attempted, in order to cut off the communication between the brain and wounded nerve.

When, however, the disease has lasted a long time, and nothing has afforded relief, and the constitution is suffering very much from the irritation, the division, or the removal of a portion of the nerve ought to be tried, or the amputation of the part where the injury was received. Amputation of the thumb has proved successful in Mr. G. Bell's case, where the nervous symptoms proceeded from a wound: it has also been successful in Mr. Wardrop's case, where the disease originated in a prick of the finger. The loss of a thumb is a very serious one, but when we consider that it is of no use in its present state, and set against the loss the agony which the patient would otherwise continue to endure if the removal of part of the nerve has not succeeded, I think the probable advantage of being freed from pain, and consequent injury of the constitution, may reasonably be looked upon as fully compensating for the loss. Should the disease be in a finger, less hesitation about its amputation would be necessary. When it is in a large nerve of the upper or lower extremity, and a question arises whether the limb should be amputated, or a portion of the nerve removed, the latter, I think, ought always to be done, except under very particular circumstances.

From the consideration of the preceding cases, it must be confessed that much remains to be desired in the treatment of these distressing accidents." 142.

Mr. Swan concludes the chapter with an allusion to Mr. Pearson's recommendation, to induce extensive cutaneous irritation in cases where the nervous system seems generally affected by the local injury. Mr. P. employs a liniment composed of olive oil, turpentine, and sulphuric acid. In the instance of a young lady, in whom the arm and hand had become nearly useless, three applications of this liniment were sufficient to effect a cure.

### VIII. Of the Effects of Ligatures on Nerves.

Passing over Mr. Swan's account of the processes which follow the application of a ligature to a nerve, and of the symptoms, which are almost obvious, we may state that he is of opinion that the application of a ligature to a nerve is scarcely justifiable under any circumstances. In cases of hæmorrhage the artery can generally be separately secured, and, if it cannot, Mr. S. thinks the actual cautery would be preferable. We feel inclined to doubt the necessity for so much dread as Mr. Swan would appear to entertain. We have seen the ulnar and the tibial nerves included on several occasions in a ligature, and have witnessed very often the tying of smaller nerves. Yet we have never seen any serious consequences follow, and the pain subsided sooner than might reasonably have been anticipated.

"When, however, the application of a ligature is decided upon, it ought to be fine, and strong enough to allow of its being drawn so tight as to completely strangulate the part of the nerve on which it is applied, for then the nerve is brought nearly to the same state as when it has been simply divided. On the contrary, if the ligature be coarse, it will not completely strangulate the nerve, consequently there will be great irritation and increase of danger to the patient. And unless the nerve be so firmly compressed by the ligature as to prevent all communication of sensation, and likewise circulation of blood between the two portions of nerve, the ligature would cause such an enlargement of both its extremities by the irritation it would create, and would be with such difficulty dislodged, that if the patient should not suffer extremely at the time, it would most probably cause the formation of a tumour, producing so much pain as gradually to wear out his strength." 151.

We fancy that few surgeons will voluntarily tie a nerve. Its ligature is usually effected in bungling attempts to secure an artery, and the thread that is employed is such as is adapted to the latter. For those who do intend to tie nerves, or expect to include them with the artery they are seeking for, Mr. Swan's injunctions respecting the ligature may be useful.

Mr. Swan quotes an interesting case from Dr. Hennen. The axillary nerves appeared to have been tied, and traction exercised upon the ligature occasioned exquisite pain referred to the course and along the distribution of the nerves of the extremity. He also alludes to two cases mentioned by Larrey. The patients died of tetanus. In one, where the arm was amputated, and the median nerve had been included in a ligature with the humeral artery, the portion of nerve below the ligature was swelled out like a mushroom, and that above was much enlarged and of a reddish colour. In the other, nineteen days after the amputation of the leg, the extremities of the nerves were swollen in the same manner, and adhering to the surrounding parts. Portal* relates a case, where this tumor formed above a ligature that was put on the sciatic nerve after amputation of the leg. The patient

* Memoires de Chirurgie Militaire, tom. iii. p. 290.

had suffered horrible pains for more than two years, which he always referred to the end of the foot; and after death this tumor was found.

### IX. On the Compression of Nerves.

Mr. Swan commences by remarking that a nerve may admit of a certain degree of extension, without uneasiness or pain resulting. But if the extension be considerable pain is excited; and if the extension be increased, the pain is increased in proportion, till at length the nerve begins to ulcerate, and if the pressure be not removed it becomes almost destroyed by this process.

"When a nerve is pressed against a bone for a short time, an uneasy sensation is produced, and the parts to which it is distributed feel benumbed. When the pressure is continued longer, these parts entirely lose the power both of sensation and motion; but if it has not been very violent they will recover.

It is difficult to say precisely in what manner a nerve is affected by pressure, when its functions are destroyed without producing any altered appearance. When there is paralysis unattended by pain it is most probable that there is merely a functional disorder of the nerve. But when the paralysis continues, and particularly if there be pain and tenderness in the part of the nerve that has been pressed, it may be suspected that inflammation has been excited and coagulable lymph effused between the fibrils, so as to produce enlargement." 156.

The treatment of nerves compressed and occasioning pain is simple. Leeches, evaporating lotions, and rest, in the first instance, succeeded if necessary, by counter-irritants, constitute the obvious remedies. Stimulating liniments and friction are required when the function is impaired, without inflammatory action being present.

"People frequently complain of pain in the lower extremities which is sometimes very excruciating. It is often occasioned by the use of tight garters, which press the nerves against the bones. In these cases it is generally absent in bed, and increases towards the latter part of the day, and has often been altogether relieved by discontinuing garters and whatever could make any undue pressure." 157.

Mr. Swan observes that where pains in the limbs are complained of, the state of the feet, and particularly that of the nails should be examined. He relates a case in illustration of this caution. The patient had intermitting pains of the great toe and ankle of the left side. There seemed to be a little thickening at one corner of the nail, and on pressing this the pain was immediately produced. Mr. S. cut off this portion of nail and found an indentation in the flesh about the size of a pin's point. This slight operation was very painful. He was very subject to disorders of the digestive organs.

Bark, an opiate draught at night, and friction of the leg with camphorated liniment, effected the patient's cure. Perhaps it might be thought that the quina divided the merits of the cure with the scissors.

Mr. Swan mentions a case in which pain in the left leg was produced and kept up by the pressure of an encysted tumor on the anterior crural nerve at its origin. Pain is often felt in a part from pressure on the origin of the nerve that supplies it. This frequently takes place in the course of the sciatic nerve, and sometimes of the anterior crural. Purging has given great relief when the pain has arisen from a distention of the sigmoid flexure of the colon and the rectum. Portal relates the case of a woman who had a very great curvature of the spine, and three or four hours after each meal complained of much pain in the great toe of the left foot; it was always increased by injections, but went off when she had a copious alvine evacuation. It was found to have been produced by pressure made by the last false ribs on the sigmoid flexure of the colon, which caused the fæces to have great difficulty in passing, and in consequence compressed the lumbar plexus of nerves.

Mr. Swan details the particulars of a case, in which an injury of the shoulder appears to have affected the axillary nerves. The patient, a lady about sixty years of age, fell from some height, and struck the head and shoulder. Mr. Swan discovered, on examination, that the portion of bone which forms the glenoid cavity of the scapula had been broken. She complained very much of pain, which struck up the neck and down to the fingers, and was very severe about the elbow; her forearm and hand were nearly paralytic. Under appropriate treatment she improved very much, and at the end of eleven weeks, the arm could be moved freely backwards and forwards without pain, though it could not be lifted very far from the side without considerable pain. She continued to advance towards recovery, till four months after the occurrence of the accident, when, the arm being suddenly and rather violently moved, very great pain was immediately produced. Mr. S. could not discover that any fresh injury was done to the bone, but all the former symptoms returned, and she was brought back to nearly her original condition. It was fourteen months after the injury before the patient had regained the perfect use of the limb. A large blister seemed to occasion benefit.

An interesting case of apparently more severe injury to the same plexus of nerves has been published by Mr. Earle. A cure would seem to have been ultimately obtained.

"I introduce the following case, because it shews that when there has been a paraplegia, that it is not merely of consequence to draw off the urine on account of the safety of the bladder, but because its over-distension presses on the nerves and tends to prevent their recovery. This idea forcibly struck me the first time I used the catheter for the patient who is the subject of the case. The bladder was very much distended, and urine kept constantly running from the penis. The distenion of the bladder caused much uneasiness; but immediately after I had drawn off two quarts of urine, the lower extremities were more sensible, and that tingling was felt in them, which is known to every one who has been in a position to press the sciatic nerve, and has experienced the sensation that arises when the pressure is removed; and I conceive that such repeated pressure on the nerves, which are already very much enfeebled, must tend very materially to retard, if not to prevent, their restoration." 165.

The preceding quotation so fully explains the fact and the inference for which Mr. Swan introduces the case, that we do not think it necessary to extract the latter. Mr. S. concludes the chapter with an instance of what he thinks was determination of blood to the spinal chord. It is practically valuable, and we fancy most practitioners must have met with some resembling it.

*Case.* The patient was a boy, aged three years and nine months. He went to bed, apparently well, on the 17th February, 1821. In the night he dreamt, and his sleep was very much disturbed. Towards morning he complained of pains in his legs, and was obliged to be taken out of bed by his mother very early. When brought to Mr. S. at 8, a.m. he had entirely lost the use of his lower extremities. His pulse was quick, his tongue furred. He had no head-ache, nor did he make any complaint of his back. Mr. Swan ordered six ounces of blood to be taken from the arm, which he found to be very florid, and a blister to be applied to the back: and two grains of submuriate of mercury to be taken every four hours. In the course of the day he moved his legs, and the next morning was able to walk. In a few days he appeared quite well, and has remained so ever since. The stools were at first quite black, but the blood on standing had not an unhealthy appearance. Whatever was the actual condition of the brain, the medulla, or its membranes, there can be no question of the propriety of the practice adopted by Mr. Swan.

Here we must bring our notice of the present work to a conclusion. The remaining space is the property of Dr. Hall and Mr. Earle. We have not been enabled to analyze or to review the last five chapters of this volume. They are dedicated to diseased appearances in the spinal canal—injuries of the spinal cord—diseases of the nerves of the senses—diseases of the sympathetic nerve—and tetanus. To these we shall advert in our next Number.

---

We now proceed to the second work upon our list—a paper on the Reflex Function of the Medulla Spinalis and Oblongata, by Dr. Marshall Hall.

The Philosophical Transactions of this Spring have furnished two or three important articles connected with medical science—one of which is that before us. It is appropriately dedicated to Mr. Brodie, whose labours in physiology and surgery generally have laid the profession under deep obligations.

In this memoir, Dr. H. proposes to give an account of a principle of action in the animal economy, which, he thinks, has not hitherto been distinguished with sufficient precision from the other vital animal functions. This principle is peculiarly connected with the medulla oblongata and spinalis. It is well known that M. Legallois considered the spinal marrow as a whole, and in distinct portions, to be the exclusive source of sensation and voluntary motion. Cruveilhier, on the other hand, denounces this view, as one of the errors of modern physiology. The reasonings of the latter author are thought to be much too exclusive by Dr. Hall. On the more recent occasion of a report on the work of M. Flourens, the Secretary of the Institute remarks, that " the author concludes that sensation and contraction appertain no more to the spinal marrow than to the nerves; and this conclusion is just, as far as unmutilated animals are concerned. It is, however, a question whether the rule will apply to those animals who have been decapitated, and which, in certain classes, are far from losing their animal functions immediately on privation of the brain."

" It was a singular mistake (says Dr. Hall) to imagine that the same conclusion could be just in reference to the entire animal, which was incorrect in reference to the animal deprived of its encephalon. The facts are these: in the entire animal, sensation and voluntary motion, functions of the cerebrum, combine with the functions of the medulla oblongata and medulla spinalis, and may therefore render it difficult or impossible to determine those which are peculiar to each; if, in an animal deprived of the brain, the spinal marrow, or the nerves supplying the muscles, be stimulated, those muscles, whether voluntary or respiratory, are equally thrown into contraction, and, it may be added, equally in the complete and in the mutilated animal; and, in the case of the nerves, equally in limbs connected with and detached from the spinal marrow.

The operation of all these various causes of muscular contraction may be designated *centric,* as taking place *at,* or at least in a direction *from,* central parts of the nervous system. But there is another function the phenomena of which are of a totally different order and obey totally different laws, being excited by causes in a situation which is *eccentric* in the nervous system, that is, distant from the nervous centres. This mode of action has not, I think, been hitherto distinctly understood by physiologists. It is involved in the question which Baron CUVIER considers as so full of interest, and is that treated of in the following pages.

Many of the phenomena of this principle of action, as they occur in the limbs, have certainly been observed. But, in the first place, this function is by no means confined to the limbs; for, whilst it imparts to each muscle its appropriate tone, and to each system of muscles its appropriate equilibrium or balance, it performs the still more important office of presiding over the orifices and terminations of each of the internal canals in the animal economy, giving to them their due form and action; and, in the second place, in the instances in which the phenomena of this function have been noticed, they have been confounded, as I have stated, with those of sensation and volition; or, if they have been distinguished from these, they have been too indefinitely denominated instinctive, or automatic. I have been compelled, therefore, to adopt some new designation for them, and I shall now give the reasons for my choice of that which is given in the title of this paper.

This property is characterized by being *excited* in its action, and *reflex* in its course: in every instance in which it is exerted, an impression made upon the extremities of certain nerves is conveyed to the medulla oblongata or the medulla spinalis, and is reflected along other nerves to parts adjacent to, or remote from, that which has received the impression.

It is by this reflex character that the function to which I have alluded is to be distinguished from every other. There are, in the animal economy, four modes of muscular action, of muscular contraction. The *first* is that designated *voluntary:* volition, originating in the cerebrum, and spontaneous in its acts, extends its influence along the spinal marrow and the motor nerves, in a *direct line,* to the voluntary muscles. The *second* is that of the *respiration:* like volition, the motive influence, in respiration passes in a *direct line* from one point of the nervous system to certain muscles; but as voluntary motion seems to originate in the cerebrum, so the respiratory motions originate in the medulla oblongata: like the voluntary motions, the motions of respiration are spontaneous; they continue, at least, after the eighth pair of nerves has been divided. The *third* kind of muscular action in the animal economy is that termed *involuntary:* it depends upon the principle of irritability, and requires the *immediate* application of a stimulus to the nervo-muscular fibre itself. These three kinds of muscular motion are well known to physiologists; and I believe they are all which have been hitherto pointed out. There is, however, a *fourth,* which subsists, in part, after the voluntary and respiratory motions have ceased, by the removal of the cerebrum and medulla oblongata, and which is attached to the medulla spinalis, ceasing itself when this is removed, and leaving the irritability undiminished. In this kind of muscular motion, the motive influence does not originate in any central part of the nervous system, but at a distance from that centre: it is neither spontaneous in its action, nor direct in its course; it is, on the contrary, *excited* by the application of appropriate stimuli, which are not, however, applied immediately to the muscular or nervo-muscular fibre, but to certain membranous parts, whence the impression is carried to the medulla, re-

*flected*, and reconducted to the part impressed, or conducted to a part remote from it, in which muscular contraction is effected." 638.

Dr. Hall proceeds to observe, that the first three modes of muscular action are known only by actual movements or muscular contractions. But the reflex function exists as a continuous muscular action, as a power presiding over organs not actually in a state of motion, preserving in some, as the glottis, an open,* in others, as the sphincters, a closed form, and in the limbs, a due degree of equilibrium, or balanced muscular action.

The critic may hint, that the instances adduced of this new kind of muscular action—of this "reflex function," are not so satisfactory as could be wished. The sphincter of the eye, or of the mouth, does not differ in kind from the sphincter of the anus. Yet the former are supplied by a nerve of voluntary motion—the portio dura (as the latter is by branches of the sacral spinal nerve)—are themselves susceptible of voluntary motion and contraction—and probably differ in no essential respect, except in anatomical disposition, from other voluntary muscles. At all events, Dr. Hall has not proved that they do so. It has long been known, that the ordinary condition of a muscle is intermediate between its extreme degrees of contraction and of relaxation. This is proved by its contractility on one hand, and its extension, by antagonizing muscles, on the other. If, then, Dr. Hall terms this condition the consequence of a "reflex function," it might be plausibly urged that he re-christens a familiar object.

"The three kinds of muscular motion hitherto known may be distinguished in another way. The muscles of voluntary motion and of respiration may be excited by stimulating the nerves which supply them, in any part of their course, whether at their source, as a part of the medulla oblongata or medulla spinalis, or exterior to the spinal canal: the muscles of involuntary motion are chiefly excited by the actual contact of stimuli. In the case of the reflex function alone, the muscles are excited by a stimulus acting mediately and indirectly in a curved and reflex course, along superficial sub-cutaneous or sub-mucous nerves proceeding to the medulla, and muscular nerves proceeding from the medulla. The first three of these causes of muscular motion may act on detached limbs or muscles. The last requires the connexion with the medulla to be preserved entire." 639.

It will be noticed that our author lays down a distinction between the nerves of respiration and those which are governed by this reflex function, say the sphincters. Again, the critic might suggest that, when narrowly examined, the distinction is not very easily drawn. The sphincter ani, for example, can be voluntarily contracted—so can the diaphragm; irritation of parts with which the sphincter is associated in function, will occasion involuntary contractions of the latter—the same law holds good with the diaphragm; pressure on the brain, or on the spinal marrow above the part which gives rise to the nerves that supply it, will cause palsy of the sphincter—so it will of the diaphragm—the sphincter ani maintains an ordinary state, intermediate between its extreme contractions which are voluntary or involuntary, and extreme dilatation which is only mechanical—the same may be said of the diaphragm, the only difference being that the ordinary state of the latter is motion, of the former, rest.

---

* See LEGALLOIS, Op. cit. p. 176—178.

Let us meet the question as openly as possible, and expose it fairly to the light of day. We will take for the subject of our observations the sphincter of the eyelids (orbicularis oculi). We remark first of all, that it maintains, like all other muscles, an ordinary condition, intermediate between the extremes of contraction and dilatation. The physiological world was sufficiently aware of this fact, and attributed it partly to the influence of the nerves supplying the muscle, partly to the properties of the muscular tissue itself. In assigning this as a consequence of the reflex function, we do not see that Dr. Hall has made much advance in the process of explanation. In the second place, we perceive that the sphincter is under the influence of the will. Now this must be the effect of *a* reflex function, though not of *the* reflex function. A frightful image is presented to the eye, an impression is made upon the brain, and the latter wills that the eye be closed. In the third place, the eyelids are seen to close convulsively when an irritating substance is applied to the surface of the cornea. This is commonly believed to be owing to an impression made upon the fifth nerve, transmitted to its origin, and giving rise to reflected action of the seventh, or at least of the muscles supplied by it. Is this Dr. Hall's reflex function? If so, it is sufficiently familiar; if not, in what does it differ from it? Dr. Hall may rejoin, that in this instance actual sensation is produced. But that is mere accident. If the fifth had not been a nerve of sensation, if the kind of irritation had not given rise to sensation at all, the mode of excited action would still have been the same. In the fourth place, we see contractions of the sphincter of the eye forming one of an associated set of actions, as in coughing or in sneezing. In this instance an impression is made upon the fifth in Schneider's membrane, or on the eighth in the larynx, and contraction of many different muscles, supplied by various motor nerves ensues. No one has ever doubted that this was the result of a reflex function, though no one, for aught we know, has employed precisely the same designation for it.

We do not wish to push the argument further. We may possibly, very probably, under-estimate the ingenious views of Dr. Hall. Yet so far as our abilities enable us to comprehend their bearing, they do little more than present an old friend with a fresh face, rouged, perhaps, a little, and his whiskers dyed, but retaining the features and expression which have been familiar.

To return to our able author. He cites an instance illustrative of the distinctions between irritability and the reflex function.

"It is important," says he, "to shew still more distinctly than I have done, the distinction between the movements arising from the reflex function of the medulla oblongata and medulla spinalis, and those arising from irritability itself. If the glottis of an animal be touched, there is an immediate contraction. If the heart be touched, the same phenomenon is observed. What is the difference between the excited movements of these two organs? If the brain be removed, the same events will take place. If the medulla oblongata be removed, the contractions of the stimulated larynx suddenly cease, whilst those of the heart continue as before. The difference consists, then, in the presence of the medulla oblongata, which is essential to the contractions of the larynx, but of which those of the heart are entirely independent. The influence of the stimulus upon the heart is immediate. That of a stimulus applied to the larynx must pass to the medulla oblongata, and be reflected upon the part moved." 643.

The sum of the "reflex function" may, perhaps, be comprised in the direct expression—that the action of those muscles supplied by cerebral or spinal nerves, depends on the preservation of their connexion with the portions of the cerebrum, or the medulla, from which those nerves originate, or to which, to use another term, they are referred.

The preservation of this property by segments of the cerebro-spinal nervous system, in the lower animals, is well exemplified by the following experiment of Dr. Hall.

"On another occasion, having removed the head of a frog, I divided the spine between the third and fourth vertebræ, and separated the upper portion of the animal from the lower. There were then the head, the anterior extremities, and the posterior extremities, with their corresponding portions of medulla, as three distinct parts of an animal. Each preserved the reflex function. On touching an eye, it was retracted, and the eyelids closed, whilst similar phenomena were observed simultaneously in the other eye. On removing the medulla, these phenomena ceased. On pinching the toe of one of the anterior extremities, the limb and the opposite limb equally moved. On removing the spinal marrow, this phenomenon also ceased. Precisely similar effects were observed in regard to the posterior extremities." 646.

Dr. Hall quotes some very interesting cases of anencephalous fœtus. The existence of motions independent of the brain and of volition, and dependent on the medulla, is well shewn by the details of those authentic facts. We may cite one related by M. Lallemand.

"J'ai vu," says he "il y a quatre ans, à l'Hôtel-Dieu, un fœtus anencéphale, à terme, ou à peu près, qui vécut trois jours. Pendant tout ce temps il poussa des cris assez forts, exerça des mouvemens de succion toutes les fois qu'il sentit quelque chose entre ses lèvres; mais on fut obligé de le nourrir avec du lait et de l'eau sucrée, parce qu'aucune nourrice ne voulait lui donner le sein. Il exécutait des mouvemens asses étundus des membres thoraciques et abdominaux. Quand on plaçait un corps étranger dans ses mains, il fléchissait les doigts comme pour le saisir; mais en général tous ses mouvemens avaient moins d'énergie que ceux d'un fœtus de même âge.

Le cerveau et le cervelet manquaient entièrement: il ne restait à la base du crâne que la moëlle allongée et la protubérance annulaire, avec l'origine des nerfs pneumo-gastrique, trifacial et optique. Le tout était recouvert par les débris des os du crâne, des méninges et de la peau." 648.

It must be obvious that the muscular motions in this instance must have been the result of impressions transmitted from the nerves to the medulla, and of actions impressed by the medulla on the muscles. Volition, in the strict sense, there could not have been, for the very satisfactory reason—that nothing existed to will.

Dr. Hall proceeds to shew that the reflex function admits of exaltation as in tetanus. In a frog made tetanic, and divided into three parts, the spasm continued to be produced in each by the application of stimulants.

The last division of the paper of our able author is devoted to those pathological facts which illustrate the reflex function. He thinks that the diseases of the nervous system may be divided into those of centric and eccentric origin. The accidents of dentition form a good instance of excitement of the reflex function. Epilepsy, asthma, chorea, tetanus, and other maladies, are cited as affording instances of morbid conditions of the reflex function.

Before we close this notice we think it only due to Dr. Hall, to lay before our readers a short summary of his inferences, which he introduces near the termination of his paper.

" Physiologists have hitherto enumerated only *three* sources or principles of muscular action,—volition, the motive influence of respiration, and irritability.

There is, however, a *fourth* source of muscular motion distinct from any of these, though not hitherto distinguished, to which I have ventured to give the designation of *the reflex.*

Volition and the motive influence of respiration are direct in their course, and spontaneous in their action; the former proceeding from the cerebrum, the latter from the medulla oblongata.

The movements of irritability are the result of the immediate application of a stimulus to the nervo-muscular fibre itself.

The reflex function is different from any of these:

It remains attached to the medulla spinalis, when the cerebrum and the medulla oblongata are removed: it is not direct like volition, or the motive power of respiration.

Its seat is the medulla generally: it ceases when the medulla is removed, leaving the irritability entire: it is not excited immediately, like the movements of irritability, but mediately, in a reflex course, through the medulla, from the part stimulated to the part moved.

In a state of health, the reflex function presides over the orifices and terminations of the internal canals, such as the glottis and the sphincters, preserving the former open, the latter closed; and it maintains the due tone of each muscle, and the due equilibrium of each system of muscles.

When excited, it gives origin to the movements observed in deglutition, or vomiting, sneezing, tenesmus, &c. The fingers passed into the pharynx of a dog, through an incision made between the thyroid cartilage and the os hyoides, excites the act of deglutition; passed over the root of the tongue or the fauces, it excites the associated actions of the muscles of the larynx and of expiration, which constitute the act of vomiting.

When morbidly augmented, it constitutes certain forms of disease, as tetanus, hydrophobia, certain forms of tremor, paralysis agitans, chorea, stammering, &c.; when diminished, it induces those forms of tremor observed when the vital powers are enfeebled.

When otherwise morbid, it occasions other forms of disease, as the convulsion, the croup-like respiration, the affection of the sphincters, observed in dentition, the various effects of intestinal irritation, &c.

The effects of the excited reflex function are sometimes observed in a part *near* that irritated, as in the eyelids in winking, in the glottis on inhaling a drop of water or particle of food, in the sphincter ani in dysentery, &c.; sometimes in parts *remote*, as in the irritation of teething when this induces strabismus, convulsion, the croup-like respiration, relaxed sphincters, &c." 659.

We will only add, that Dr. Hall has pointed out much more clearly than has hitherto been done the continuance of certain actions in voluntary muscles after volition is removed, and their dependence on that portion of the nervous centre with which they are immediately connected through the medium of their nerves. We cordially bear testimony to that ingenious spirit which has elucidated many questions in pathology and physiology, and we trust that Dr. Hall will continue to favour the profession with the prosecution of his inquiries, and the communication of their results.

---

We now arrive at the work of Mr. Earle, a relation of the gentleman who occupies with credit the situation of surgeon to Bartholomew's Hospital. It is termed a New Exposition of the Functions of the Nerves—a title calculated to arrest attention, and displaying the confidence and boldness of its author.

He appears to anticipate opposition to his views, but apparently possessed of an intellect superior to his age, he rises, like light hydro-carbon, through the muddy waters that surround him, and looks for appreciation and for fame to posterity. It is pleasant to observe this philosophical reliance on the taste and the judgment of our children, and we trust they will answer the reasonable expectations entertained of them.

"If the discovery of Harvey," says Mr. Earle, "admitting of such clear and satisfactory demonstration, met with violent opponents and a tardy acquiescence, in inducing facts which are addressed to the eye of reason only, I cannot expect to escape that share of prejudice and opposition which has always attended the announcement of novelty; nor that the opinions derived from the facts which I have brought forward will be readily allowed to be just; for, as it has been well observed by an old writer, 'before truth in its silent or disputed march has roused the attention of the indolent, converted the supercilious, subdued the interested and obstinate, and reached the ears of all, an age has passed away.'" 3.

The calm observer of the actions and passions of mankind has frequent opportunities of remarking the benevolent arrangements of Providence. Sources of consolation eternally spring up in circumstances the most unpromising, amidst prospects the most cheerless. The dying criminal, beneath the tree, looks back upon his crimes with scarcely a shudder, and almost ventures to anticipate hereafter the glorious fruit of his few hours' trust in his Redeemer. The literary world enjoys its revelation. Its great in guilt, and its poor in spirit, are soothed with the hope of a future state, when penitence may save, or suffering beatify them. The stripes of the critical centurion will have been effaced, the mockery of the insolent rabble will be hushed.

Could the gentle Harvey look down from his immortal habitation, he would smile with satisfaction to remark how many persecuted authors his treatment and his triumph have been the means of pacifying and of comforting. Yet a sigh might possibly escape him, on reflecting how few had their just aspirations gratified, or received from posterity the recompence that they deserved. He would find on examination of the rolls in the record office of fame, that of millions who received contemporary slander, scarce one was crowned with posthumous glory. He would probably own, that the possession of the former afforded an imperfect guarantee for the acquisition of the latter. The scoffer might hint to the ardent author the possibility of the fate of that still fameless poet; to be remembered when Homer and Virgil are forgotten, but not before.

Should our want of judgment, our indolence, or our obstinacy induce us to demur to the reasonings of Mr. Earle, and lead us to contemn or undervalue his discoveries, we possess the pleasing reflection, that he looks to a future and a better æra, for a more correct and more favourable estimate. That sympathetic powder will readily dry up the critic's wound.

The earliest philosophers found no difficulty in determining that the causes of things are buried in obscurity. Philosophers, ancient and modern, would be puzzled to determine the cause of the present book. Know, reader, it was the frequency of hydrophobia in the Spring and Autumn of 1830. The chain of causation is complete and obvious.

Averting our eyes from a painful spectacle displayed in the first twenty-four pages of the volume (we allude to the immolation of Dr. Wilson Philip), we arrive at the second chapter, devoted to the new exposition of the nature of nervous influence, and the classification of the nerves. Curiosity is naturally roused to learn the great truths, which, however neglected in this age, may constitute the wealth of succeeding centuries.

"I shall now proceed to state the grounds upon which I consider myself justified in believing that a something, which has been called Nervous Influence, is constantly emanating from the brain; and then by classifying the several sorts of nerves according to the functions which each performs, to explain the mode in which this single power is made use of for the accomplishment of every purpose relating to the economy of animal life." 25.

It must be owned that this idea does not appear, on a superficial examination, to be new, but closely to resemble an old and an exploded theory. But Mr. Earle proceeds to remark, that many deny the existence of any fluid emanating from the brain and passing along the nerves, on account of its being invisible. He refers to the electric organs of the torpedo, as affording a proof of the justice of his theory. After exertion of those organs, the electric power appears to be exhausted, and requires rest for its renewal. Yet rest alone seems inadequate, in Mr. Earle's eyes, to repair the electric power, which he therefore supposes to be renovated in the brain. We confess that we think Mr. Earle has travelled farther than he need have done. The nerves of the senses, in all animals, are fatigued by long-continued exercise, and we see no difference between the two cases. One proves as much and as little as the other. Mr. Earle, however, shouts—Io triumphe!

"If there are any who still deny the possibility of there being a fluid which comes from the brain and passes along the nerves, because it cannot be rendered evident to the sight, of them it may be demanded, what difference does the eye perceive between oxygen and nitrogen gas, or between hydrogen and carbonic acid? Neither of these gases have the same properties, though they are all equally fluid and invisible. Away then with the puerile objection of those who ask, who ever saw nervous influence? Is the caloric visible in the mercury of a thermometer, when its column stands at the boiling point?" 29.

No doubt the analogies are forcible, but we do not quite perceive their connexion with the matter. As it is not our object to argue much with Mr. Earle, we shall do little more than lay his views before our readers.

He observes that it is generally admitted, that the influence of the brain is necessary to secretion, and, by a piece of logical harlequinade, he immediately concludes that this secernent power resides in the whole brain, while volition and sensation belong only to parts of it. This pantomimic leap is dexterous, and should be shewn.

"I consider this secernent function to be the result of an action of the whole brain for two reasons, first, because it is proved by Dr. Philip* that the action

* Experimental Inquiry into the Laws of the Vital Functions.

of the heart cannot be affected by a stimulant, however intense, if it be applied to a small part only, but the motion of this organ is immediately increased when a stimulant is applied to any part of the brain, provided the extent of the surface stimulated is considerable ; secondly, the size of the brain is so small as compared with that of the body, that I think it is scarcely possible to be supposed that an action of a part only of this organ could be equal to the maintenance of all the vital functions. While the secernent function which maintains the health of the body is constant and uniform, and belongs to the whole brain, we have every reason to believe that volition and sensation, which provide for its convenience and welfare, belong only to particular parts of it, and are not always engaged." 32.

We think the above a fair specimen of our author's style of reasoning, which appears to consist in a very liberal employment of that useful mode of argument —the petitio principii. He proceeds to state that, as the brain is constantly acting, and the nerves are the only means by which that action can be made available, the manner in which the latter perform their office becomes a fit subject of inquiry. On this head experiments, of course, are necessary. But the results obtained from the division of a nerve containing many filaments are inconclusive. Thus, says Mr. Earle, the division of the eighth pair of nerves in the neck is followed by a loss of the secreting power in the stomach ; but that does not inform us of the mode in which the blood had acted upon the blood in the vessels of the stomach, to produce secretion:—a conclusion which, we fancy, will be readily admitted. In order to arrive at such desirable information as that pointed out by Mr. Earle, it is necessary, says he, to inquire how many sorts of filaments nerves are composed of, and then to ascertain what are their separate modes of action. We shall feel great pleasure, and possibly some surprise, if Mr. E. is enabled to do this.

After noticing Dr Philip's classification of the nerves, and pointing out its defects and incongruities, Mr. Earle proposes the following as the principle which guides his own.

" I venture to propose that their different functions, according to what is at present known of them, should form the basis of their classification, and that they should be considered, first, according to the functions which each performs independently of the others, and secondly, according to their natural arrangement and the functions which they perform in combination. I do not propose this natural classification of the nerves merely on account of its novelty, but on account of the real advantages which it possesses. It will be found to render that which has hitherto been enveloped in the darkest obscurity at once clear and satisfactory ; it will also introduce a degree of precision hitherto unknown in physiology, and thus remove the discredit into which this branch of medical science has fallen." 38.

We must now introduce a rather long quotation, in order to exhibit our author's arrangement in his own words. We should mention that the Italics and the capitals are his property, not our interpolations.

" In unison with my plan of arranging the nerves according to their separate functions, I propose, that the term cerebral nerve, instead of as hitherto being applied to every one which passes through an aperture in the skull, should be restricted to the olfactory, ophthalmic, and auditory nerves, on account of their being more particularly connected with the intellectual functions which are now generally regarded by physiologists as performed in some way or other by the brain, and from their being the most simple in their function, which is only that of transmitting impressions from their extremities to the brain, and from their filaments not being intermingled with those of any other nerve, that they should form the FIRST CLASS, and be considered as the only purely *cerebral* nerves.

That the SECOND CLASS should comprehend every nerve by means of which muscles are subjected to the influence of volition, these are the anterior nerves, and belong to the anterior columns of the spinal marrow. As this division embraces the third, the fourth, the anterior root of the fifth, the sixth, the portio dura of the seventh, and the ninth nerves, it will be seen that I have partially adopted the classification of Sir C. Bell, and, consequently, consider all the medullary fibres below the first formation of the crura cerebri, as comprised under the term Anterior Columns of the spinal marrow, without regard to the circumstance of those fibres being encompassed at a particular part by other bands of medullary substance, termed pons varolii; or separated by other bodies which have been termed corpora olivaria.

That the THIRD CLASS should comprehend every nerve belonging to the Posterior Columns of the spinal marrow; thus including the posterior root of the fifth, the glosso-pharyngeal the pneumogastric and Spinal Accessory nerves, all of which, with the exception of the last, transmit impressions from their extremities to the brain, and have protuberances upon them just before their respective junctions with the posterior columns of the spinal marrow; by the term Posterior Columns is signified their whole extent as far as the terminations of the restiform bodies in the posterior peduncles of the cerebellum. The ganglions on these posterior nerves will, in the course of these observations, always be distinguished from those of the sympathetic by the term posterior ganglions, for the sake of avoiding any misunderstanding.

Lastly, that the FOURTH CLASS should comprehend every nerve proceeding from the sympathetic ganglions.

Every part to which branches of these nerves are distributed, whether its structure is muscular as that of the heart, or fibrous as that of the arteries and iris, has a power of motion altogether independent of any influence that the will can exercise.

Such is the classification which I consider the distinct nature of the nerves obviously points out, and which I think is more particularly warranted by the well-known fact that nervous filaments, however intermingled, whether united with others near to the brain and spinal marrow, or at a distance from them, never change their character. I must particularly beg the reader's attention to this arrangement, because it will enable him to understand more readily the observations I shall have to make respecting the functions of the nerves, and the effects resulting from their combination.

These four classes of nerves are found distributed in four different orders.

The First Class, or purely cerebral nerves, are the most simple in their arrangement, because their filaments are never intermingled with those of any other nerve. These therefore should be considered as forming the FIRST ORDER OF DISTRIBUTION.

The next most simple arrangement is found in those nerves which are formed by the union of anterior and posterior filaments, or *the second and third classes.* These therefore should form the SECOND ORDER. These nerves supply the bones, muscles, &c. of the limbs and a great part of the trunk of the body; and those parts, whose structure is such as to allow of motion, can at any time be called into action by the will.

The next variation is found in the union of posterior filaments with those belonging to sympathetic ganglions, or the *third and fourth classes.* These, therefore should form the THIRD ORDER. All parts supplied by these nerves, whose structure is such as to allow of motion, such as the heart, arteries, intestines, and iris, are observed to be in constant action from birth till death, without becoming exhausted or fatigued and without requiring rest. The action of these parts cannot be affected by the will.

The last variation is found in those nerves which are formed by the union of anterior, posterior and sympathetic filaments, *or second, third, and fourth classes.* These, therefore, should form the FOURTH ORDER. The muscles supplied by filaments arranged in this order are observed to have a power of supporting constant motion without fatigue, but they differ from those supplied by the third order in being always obedient to the commands of the will. The nerves and function of the diaphragm afford an example of this variety." 43.

A critic, as severe towards Mr. Earle as he has been to Dr. Philip, would readily enumerate some striking defects in the foregoing classification. A practical physiologist might contemn the whole—might demand with a sneer, to what it amounted—and sarcastically observe, that it resembled the dispute between the Babylonian sects, respecting the necessity of putting the right or the left foot foremost on entering the temple of Baal. But we waive such criticism and disclaim such censoriousness, contenting ourselves with placing, as far as our crippled space will permit, the peculiar views of Mr. Earle before the public.

Mr. Earle would seem to have discovered, with little difficulty, the use of the spinal accessory nerve. As this has perplexed, if it has not baffled, very able physiologists, the facility with which it is determined by our author affords a striking instance of the value of his views.

"It is by means of this nerve that the trapezius and mastoid muscles are enabled to assist in supporting and moving the weight of the head for a great length of time without becoming fatigued." 45.

We formerly hinted at the doctrine of a circulation of the nervous system entertained by Mr. Earle. The course of that circulation is thus laid down by him.

"It now only remains that I should state the precise manner in which I consider the influence of the brain, or more properly the CEREBRAL INFLUENCE, because it proceeds from the cerebrum, and not from the cerebellum, is applied by the nerves in order to effect secretion, and to supply the voluntary and involuntary muscles with their power of contraction. The anterior and posterior nerves, or second and third classes, bear the same opposite relation to each other as has been already noticed with regard to volition and sensation, the one being active, the other passive or recipient. The action of the anterior nerves is always from the cerebrum towards their extremities, that of the posterior is exactly the reverse, being from their extremities towards the cerebellum. There is no instance in the whole body of an anterior nerve not being joined to a posterior nerve. This arrangement appears to be for the purpose of allowing the cerebral influence, which flows along the anterior, to return in a reflux direction along the posterior nerves and posterior columns of the spinal marrow to the cerebellum; thus completing a circulation as perfect as that which is carried on by the arteries and veins, and which I venture to call the CIRCULATION of the NERVOUS SYSTEM." 51.

This nervous circulation is productive of great consequence, for by it the "cerebral influence" regulates secretion, in the following simple and satisfactory manner.

"It appears to me that the passage of the cerebral influence, from the extremity of an anterior to that of a posterior nerve, offers a much stronger analogy to galvanism than any which has been pointed out by Dr. Philip, though I am still far from thinking it sufficient to establish their identity. It is to this passage of the cerebral influence, from the extremity of one nerve to that of another, analogous to the passing of galvanism from the positive to the negative pole, that I am inclined to attribute the maintenance of the blood in its fluid state, the evolution of caloric, the secretion of synovia in the joints, and bursæ, and, in fact, the support of the whole secernent function upon which the deposition of new, and the removal of old parts depends. It is farther to be observed, that as this circulation is constantly going on, there is always a certain quantity of cerebral influence in the anterior nerves, consequently all the muscles to which these nerves are distributed must always act in obedience to the will, simply because volition can effect their prime origins, which are in the cerebrum." 52.

As none can resist the force of truths so weighty and of demonstration so complete, it would be idle in us to offer any observations in opposition to them, or in confirmation of them. We have only to remark that, as novelty and ingenuity are displayed in every page, and as not only new views but a new mode of reasoning, new, at least, since induction became fashionable, are presented, we find that it is hopeless to attempt a consistent account of this work of Mr. Earle's. We therefore fear we must content ourselves with one more quotation, which, brief as it may be, contains the nucleus of more originality than many ponderous volumes. It is difficult to avoid the expression of astonishment at the depth of penetration and soundness of judgment displayed by the able author. It is impossible to repress a feeling of exultation that such discoveries, however ridiculed they may be by fools, will reflect a glory, in future times, upon the present century.

" In this simple arrangement, however, there is no provision for the supply of the viscera, but as their secernent and motor function equally require the presence of cerebral influence, there must be some contrivance whereby it may be supplied. For the accomplishment of this object, every posterior nerve is provided with a ganglion, (which as they do not interfere with the transmission of external impressions, evidently perform some office which has not hitherto been understood,) in order that part of the current which is returning in a reflux direction along the posterior nerves, upon which alone these protuberances are found, may pass at regular intervals into the sympathetic ganglions. How apparent the object of the wandering course of the pneumogastric nerve (which is entirely composed of posterior filaments at its termination, or what has hitherto been considered its origin,) now becomes? The secernent function of the viscera must be performed in the same way as in the limbs, and an opportunity is thus afforded by the pneumogastric nerves, for the passage of the same fluid from the extremities of the nerves coming from sympathetic ganglions, to the extremities of nerves of an opposite character, at once providing for the whole secernent function of the viscera; the differences in the various secretions not resulting, except in some degree perhaps in the liver, from any difference in the blood itself or in the fluid which acts upon it, but from the peculiarity of the structure of each secreting organ. I have no words to express my admiration of the beauty of this arrangement, whereby the motion of all parts supplied by the third order of nerves is rendered both involuntary and unceasing: involuntary because the influence of the will cannot possibly extend beyond the extremities of the anterior nerves, and unceasing because that which bestows the power of motion is supplied in an uninterrupted stream. Are we now able to explain the reason why the heart has nerves? and why every variety of mental emotion has an effect upon secretion in proportion to its intensity?" 53.

How clear is the light which emanates from the mind of the man of genius. We may now explain or comprehend the reason why the heart has nerves, a great truth which was heretofore utterly incomprehensible—and why we cannot make our viscera contract like our limbs. How beautiful is the system of the ganglia—how wonderful is its discovery. If we lay aside the pen, it is not because it cannot be worthily wielded in spreading the knowledge of the labours of Mr. Earle—it is not because a feeling of fatigue may provoke us to descend from flights of imagination so exalted, or the painful pursuit of subtleties so recondite—it is not because we are disgusted with the attempt at exposing endless fallacies, or disproving assumptions which make no pretence to proof—it is not for such reasons which uncharitable persons might deem sufficient—but it is because we feel how impossible it would be, with our limited space, to do justice to the fertile fancy and the new philosophy of Mr. Earle, and because we would wish those who may desire to be well acquainted with its contents to study the volume themselves. We can only say that singular as was its origin, a hydrophobia panic, it is worthy of a source of such rational interest.

